# Applying Supercritical Fluid Technology to Prepare Ibuprofen Solid Dispersions with Improved Oral Bioavailability

**DOI:** 10.3390/pharmaceutics11020067

**Published:** 2019-02-03

**Authors:** Fei Han, Wei Zhang, Ying Wang, Ziyue Xi, Lu Chen, Sanming Li, Lu Xu

**Affiliations:** 1 School of Pharmacy, Shenyang Pharmaceutical University, Shenyang 110016, China; hanfei_spu@163.com (F.H.); zhangwei9501@126.com (W.Z.); lnxiziyue@163.com (Z.X.); chenlu182125@163.com (L.C.); lisanming2018@163.com (S.L.); 2 Key Laboratory of Structure-Based Drug Design & Discovery of Ministry of Education, Shenyang Pharmaceutical University, Shenyang 110016, China; wangying7061blus@163.com; 3 School of Traditional Chinese Materia Medica, Shenyang Pharmaceutical University, Shenyang 110016, China

**Keywords:** ibuprofen, supercritical fluid, solid dispersion, bioavailability

## Abstract

In this study, supercritical fluid (SCF) technology was applied to prepare reliable solid dispersions of pharmaceutical compounds with limited bioavailability using ibuprofen (IBU) as a model compound. Solid-state characterization of the dispersions was conducted by differential scanning calorimetry (DSC), powder X-ray diffraction (PXRD), and scanning electron microscopy (SEM). The PXRD and DSC results suggested that the amorphous form of IBU was maintained in the solid dispersions. Furthermore, in vitro dissolution and in vivo pharmacokinetic (PK) studies in rats were also performed. The dissolution performance of the SCF-prepared IBU dispersions was significantly improved compared to that of the physical mixtures of crystalline IBU and a polymer. In addition, the PK results revealed that the SCF-prepared IBU dispersions produced remarkably high blood drug concentrations (both the AUC and C_max_) and a rapid absorption rate (T_max_). Finally, molecular modeling was used to evaluate the binding energy of interactions between IBU and the polymers. The negative binding energy suggests a relatively stable system. Hence, SCF technology can be used as a very effective approach to prepare IBU solid dispersions with good physical stability and enhanced in vitro and in vivo performance.

## 1. Introduction

Ibuprofen (IBU), a poorly water-soluble drug, is widely used as one of the best-tolerated nonsteroidal anti-inflammatory drugs for treatment of rheumatoid arthritis, osteoarthritis, and mild to moderate pain [1]. However, the poor aqueous solubility of IBU significantly limits its bioavailability and therapeutic activity [2]. In recent years, many approaches have been applied to improve solubility and bioavailability, such as encapsulation into liposomes, nanoparticles, co-milling, solid dispersion, nano-emulsification, etc. [3,4,5]. Among them, amorphous solid dispersions (ASDs) have been extensively used due to their superior solubilization [6,7,8,9,10]. Supercritical fluid (SCF) technology, a way to prepare ASDs, exhibits many unique advantages, including mild preparation conditions, environmental friendliness, controllable processing conditions, and good reproducibility. In particular, rapid expansion of a supercritical solution does not require an organic solvent as a mixing medium and can directly produce microparticles even without milling. Using supercritical carbon dioxide (scCO_2_) as a medium to prepare artemisinin dispersions can reportedly enhance the drug’s in vitro dissolution rate and intestinal absorption [11]. A solid dispersion of oxeglitazar with improved dissolution kinetics was prepared using supercritical antisolvent and spray-freezing techniques [12]. An SCF anti-solvent process was also applied to prepare telmisartan solid dispersions (SDs) using polyvinylpyrrolidone and hydroxypropyl methylcellulose as carriers [13]. Compared to conventional methods, and owing to these advantages, the use of SCF technology avoids the toxicity and pollution problems related to organic solvent usage and reduces the risk of high-energy-induced phase transitions. SCF technology with scCO_2_ as media can easily eliminate and recover the final product by controlling the pressure, temperature, aspect ratio, etc. [14]. The Kollidon grades are manufactured by a polymerization process in water without any organic solvents to form a popcorn polymer. A Kollidon system, crosslinked polyvinylpyrrolidone, including Kollidon VA 64, Kollidon CL, Kollidon CL-SF, Kollidon SR, etc. has been widely used to prepare SDs and improve oral bioavailability [15,16]. However, studies on the use of Kollidon system SDs on improving oral adsorption and bioavailability via an SCF method are still rare.

In this study, SCF technology was applied as a solvent-free manufacturing approach to prepare SDs of IBU with Kollidon system-based polymeric carriers at the lab scale. Kollidon CL and Kollidon CL-SF were selected as model polymers in the dispersions because they are reportedly good candidates for use as drug carriers and superdisintegrants in SDs to improve the dissolution rate for immediate-release dosage forms. The optimal conditions (i.e., temperature, pressure, and reaction time) for using SCF technology to prepare SDs were investigated. The solid-state forms of IBU in the SDs were investigated using powder X-ray diffraction (PXRD) and differential scanning calorimetry (DSC). Molecular modeling was also applied to predict the binding energy, which is valuable for evaluating and analyzing the interaction between SDs and poorly water-soluble drugs. Furthermore, both in vitro dissolution tests and in vivo pharmacokinetic studies in rats were conducted. The in vitro and in vivo performance of the SCF-prepared ASDs was shown to be superior to that of a drug–polymer physical mixture (PM).

## 2. Materials and Methods

### 2.1. Materials

IBU was obtained from Knoll Co. Ltd. (Purity >98%, Bad Saulgau, BW, Germany). Kollidon CL and Kollidon CL-SF (The volume average diameter was 110–130 μm and 10–30 μm, respectively) were obtained from BASF AG (Ludwigshafen, Germany). Industrial-grade carbon dioxide was purchased from Jingquan Gas Factory (Purity 99%; Shenyang, China). Deionized water was prepared by ion exchange. All other chemicals were of analytical grade and used as required without further purification.

### 2.2. Preparation of SDs

**Scheme 1:**
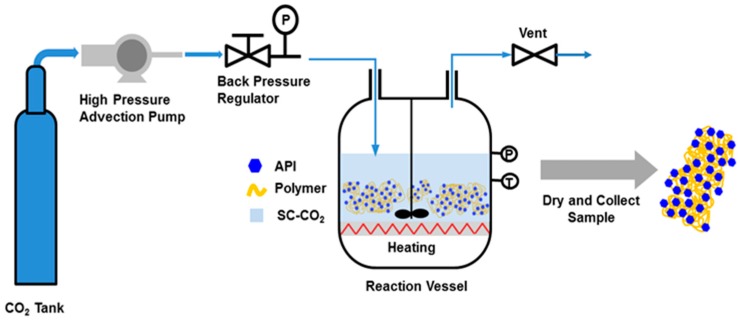
Flow chart of supercritical fluid technology.

IBU-Kollidon SDs were prepared using an SCF instrument (Shenyang Dongyu Supercritical Extraction Co. Ltd., Shenyang, China). The PMs in different ratios were placed in an scCO_2_-permeable bag, and the bag was then placed in the high-pressure vessel of the SCF instrument and processed, as shown in Scheme 1. When the vessel was closed and sealed, sufficient CO_2_ was pumped into the vessel to reach 25 MPa at 40 °C. These reaction conditions had to be maintained for at least 18 h to enable sufficient mixing of the drug and polymer in the scCO_2_ medium. Next, CO_2_ was gradually discharged from the vessel through a valve. The solid powders of IBU-Kollidon CL and IBU-Kollidon CL-SF (Named as IBU-CL SD and IBU-CL-SF SD) were collected and dried in an oven at 40 °C for at least 24 h.

### 2.3. Scanning Electron Microscopy (SEM)

SEM images were obtained using a scanning electron microscope (Type SM-T20, JEOL, Tokyo,, Japan) to analyze the surface morphology of the particles. Samples (pure IBU, IBU-Kollidon PMs, and IBU-Kollidon SDs) were mounted on metal stubs using double-sided adhesive tape and sputtered with a thin layer of gold under the vacuum.

### 2.4. Differential Scanning Calorimetry (DSC)

Thermal analysis of the IBU SDs was performed using a differential scanning calorimeter (Q1000, TA Instruments, New Castle, DE, USA). Approximately 5 mg of a sample was placed in an aluminum pan and heated from 25 to 150 °C at 10 °C/min to obtain DSC curves.

### 2.5. Powder X-Ray Diffraction (PXRD)

The X-ray diffraction (XRD) patterns of SD samples, the pure drug, and PMs samples stressed at 25 °C and 65% RH were measured by a powder X-ray diffractometer (X’pert PRO, PANalytical B.V., Almelo, The Netherlands) equipped with a Cu Kα radiation source. The experiments were conducted under ambient conditions in the Bragg–Brentano geometry at 2θ values ranging from 5° to 45° in 0.01° steps. The voltage and current were set to 40 kV and 20 mA, respectively.

### 2.6. Dissolution Studies

Dissolution tests of the SD samples were performed using a dissolution test machine (Shanghai Huanghai Medicament Test Instrument Factory, Shanghai, China) applying a USP II dissolution apparatus [17,18]. The paddle rotation speed and temperature were fixed at 50 rpm and 37 ± 0.5 °C, respectively. SDs containing the equivalent of 100 mg of IBU were placed in 900 mL of distilled water. Samples (5 mL) of the dissolution medium were withdrawn at predetermined time intervals (1, 2, 3, 4, 5, 10, 15, 20, 30, 45, 60, 90, and 120 min), and each was compensated by adding 5 mL of the corresponding fresh buffer. Next, the samples were filtered through a 0.45 μm membrane filter and analyzed by high-performance liquid chromatography (HPLC) at 220 nm.

### 2.7. Pharmacokinetic Studies in Rats

Animals were bought from Laboratory Animal Center of Shenyang Pharmaceutical University and the guidelines were approved by Shenyang Pharmaceutical University completely. Ten male Wistar rats (200 ± 20 g) were fasted for 12 h but allowed free access to water. They were divided randomly and equally into two groups for intragastric administration; one group received an IBU-CL-SF PM equivalent to 25 mg/kg of IBU, and the other received an IBU-CL-SF SD equivalent to 25 mg/kg of IBU prepared by SCF methods [19]. After ether anesthesia, 0.5 mL of blood was collected from the retro-orbital plexus at 5, 10, 15, 30, 45, 60, 90, 120, 240, and 360 min after dosing and transferred immediately to a heparinized centrifuge tube. All blood samples were centrifuged at 10,000 g for 10 min to obtain the plasma, which was transferred to a centrifuge tube and stored at –20 °C until analysis.

Plasma samples were processed as follows: exactly 0.2 mL of a plasma sample was transferred to a 1.5 mL plastic tube with a stopper and mixed with 0.1 mL of an internal standard solution (cinnamic acid, 38.1 µg/mL), 0.1 mL of methanol, and 0.2 mL of acetonitrile. After vortexing for 5 min, the mixture was further centrifuged at 10,000 g for 10 min, and a 20 µL aliquot was analyzed using a HPLC system. 

### 2.8. HPLC Analysis of IBU

HPLC analyses were performed using a Shimadzu HPLC system (Kyoto, Japan) equipped with an LC-10AT pump, a UV detector (PD-10AV) at 220 nm, and an Inertsil ODS-3 reverse-phase column (5 µm, 46 mm × 150 mm, GL Science Inc., Tokyo, Japan) maintained at 35 °C. The mobile phase was methanol/phosphate buffer (pH 3.0) solution (75:25, *v*/*v*), and the flow rate was 1.0 mL/min.

### 2.9. Molecular Docking

The 3D structures of both IBU and the polymer were built using the SYBYL 6.9.1 software package (Tripos Inc. St. Louis, MO, USA). The optimal parameters were as follows: The maximum number of iterations was 10,000, and the change in energy was 0.005 kcal/(mol × Å). 

AutoDock Tools (1.5.4) were applied to transfer both the polymer and the molecule into PDBQT format and define the binding site for a further molecular docking study. The docking results were analyzed using the docking conformations with the highest percentage frequency and the lowest binding energy as representatives.

The optimized parameters of the AutoDock 4.0 software were as follows: the maximum number of energy evaluations was increased to 25,000,000 per run, the iterations of the Solis and Wets local search were 3000, the number of individuals in a population was 300, and the number of generations was 100. Results differing by <2 Å in a positional root mean square deviation were clustered together. In each group, the lowest binding energy configuration with the highest percentage frequency was selected as the group representative. All other parameters were maintained at the default values [20].

Discovery Studio Visualizer 4.5 (BIOVIA) was used for molecular interaction analysis. 

### 2.10. Statistical Analysis

The unpaired student’s t-text was applied to do a statistical analysis. Data were presented as mean ± SD and the significant level was set at a probability of *p* < 0.001. 

## 3. Results

### 3.1. Optimization of Preparation Conditions

**Figure 1:**
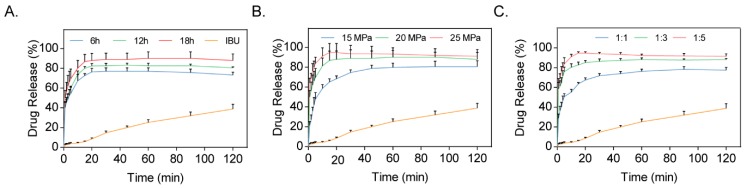
Dissolution profiles of Ibuprofen Kollidon CL-SF solid dispersion (IBU-CL-SF SD) systems prepared by the supercritical fluid (SCF) method with different preparation times (**A**), preparation pressure (**B**), and ratio (**C**).

SDs of IBU-CL-SF were prepared at different conditions and the same temperature by applying SCF technology, as shown in Table 1. The drug release profiles of SDs (Figure 1) indicated that the cumulative percentage of drug release in the IBU-CL-SF SD increased with increasing preparation time, pressure, and drug-to-carrier ratio. Both Group 5 and Group 7 had a relative higher drug release percentage. However, IBU in scCO_2_ exhibited higher solubility under a higher preparation pressure, resulting in better interaction between the drug and carriers to yield the desired SDs. Considering all the factors, the IBU-CL-SF SDs were prepared at a drug-to-carrier ratio of 1:5, a reaction pressure of 25 MPa, and a reaction time of 18 h.

### 3.2. Characterization

#### 3.2.1. SEM Results

**Figure 2:**
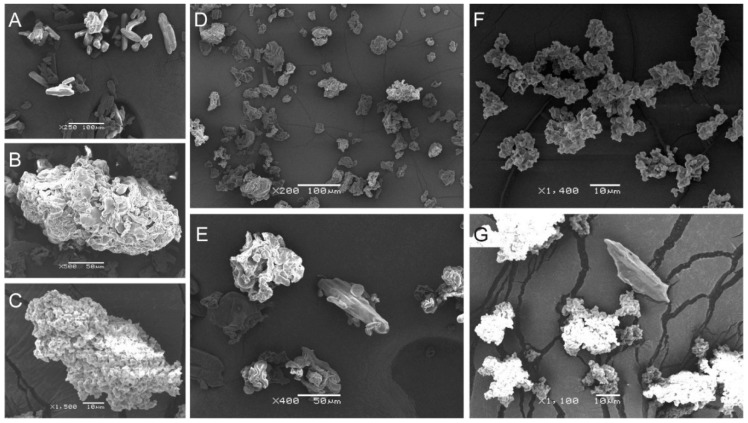
SEM images of samples. (**A**) IBU; (**B**) Kollidon CL; (**C**) Kollidon CL-SF; (**D**) IBU-CL SD; (**E**) IBU-CL physical mixture (PM); (**F**) IBU-CL-SF SD. (**G**) IBU-CL-SF PM. Scale bar is shown in the graph.

The SEM images in Figure 2 show the surface morphology of pure IBU and Kollidon, together with that of the IBU-Kollidon PMs and SDs. As shown in Figure 2A,B,E, the IBU powder exhibited rod-shaped crystals, whereas Kollidon CL and Kollidon CL-SF were observed to have rough surfaces and irregularly shaped agglomerates of different sizes. Notably, free crystals of IBU were clearly observed in the PMs but not in the two SDs, which demonstrated that IBU in the amorphous state was present in the IBU-CL and IBU-CL-SF SDs prepared by the SCF method.

#### 3.2.2. PXRD Analysis

**Figure 3:**
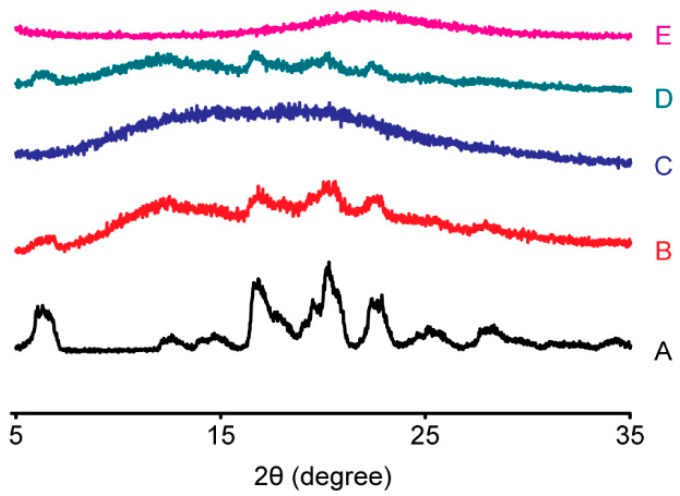
Powder X-ray diffraction patterns of the IBU-Kollidon different grade PM and SD systems prepared by the SCF method. (**A**) IBU; (**B**) IBU-CL PM; (**C**) IBU-CL SD; (**D**) IBU-CL-SF PM; (**E**) IBU-CL-SF SD.

As a complementary method, PXRD can provide direct evidence of IBU crystallization. Powder X-ray diffractograms of pure IBU and the IBU-Kollidon PM and SD systems are shown in Figure 3. Characteristic diffraction peaks of IBU were clearly observed in the IBU-Kollidon PMs but not in the SDs, suggesting that IBU was transformed into an amorphous state in the SDs of IBU-CL and IBU-CL-SF obtained by the SCF method, which is in agreement with the DSC study.

#### 3.2.3. DSC Analysis

**Figure 4:**
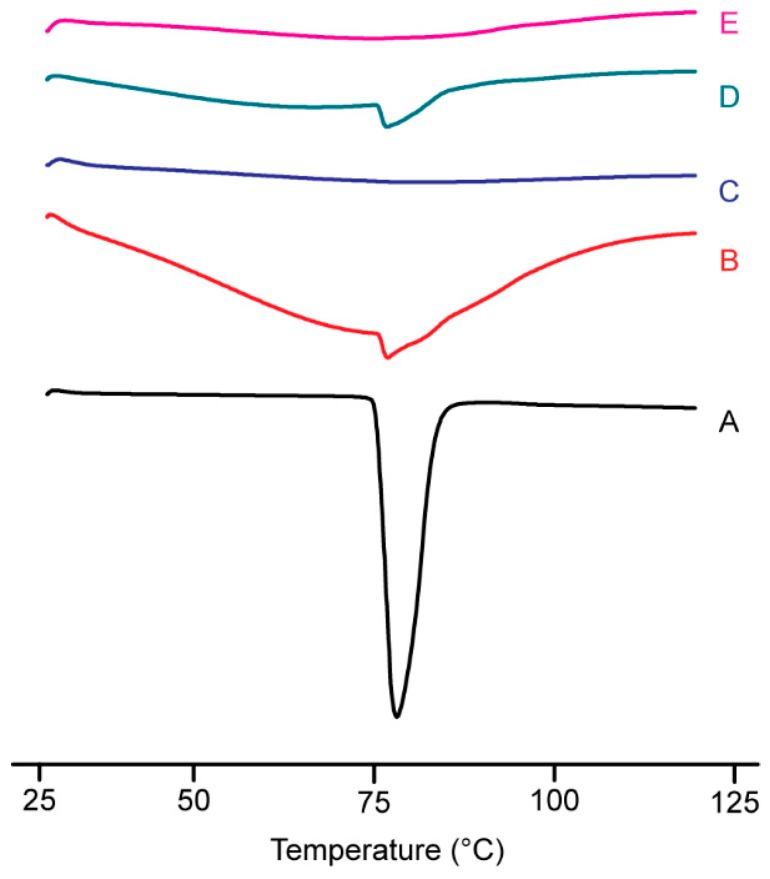
Differential scanning calorimetry curves of IBU-CL different grade PM and SD systems prepared by the SCF method (**A**) IBU; (**B**) IBU-CL PM; (**C**) IBU-CL SD; (**D**) IBU-CL-SF PM; (**E**) IBU-CL-SF SD.

To demonstrate the presence or absence of the crystalline drug, a DSC analysis was conducted. DSC thermograms of pure IBU and SDs composed of IBU-Kollidon obtained by the SCF method are shown in Figure 4. IBU is a crystalline compound exhibiting a single, sharp endothermic peak at 80.3 °C, which corresponds to the melting point of IBU. An endothermic peak was detected in the PMs, confirming the existence of a crystalline phase, although the endothermic behavior was weaker than that of pure IBU. This was in contrast to its crystalline phase in the PMs, in which the two SDs exhibited the amorphous state instead. Therefore, the drug–carrier interactions appeared to be effective for inhibiting drug crystallization.

### 3.3. Dissolution Studies

**Figure 5:**
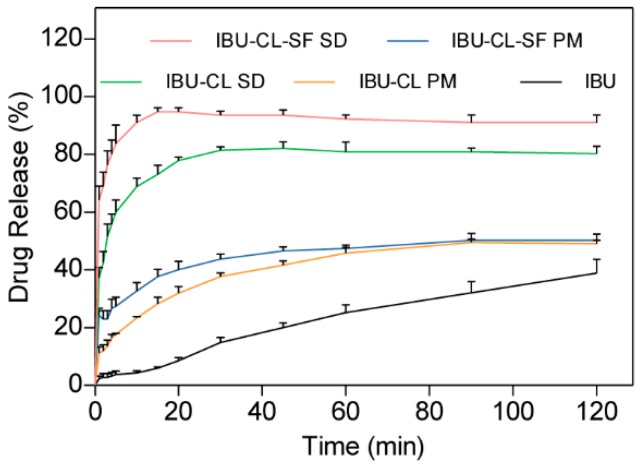
Dissolution profiles of IBU-Kollidon different grade SD systems prepared by the SCF method.

The dissolution profiles of IBU in the PMs and SDs prepared with different carriers (Kollidon CL and Kollidon CL-SF) were investigated and are shown in Figure 5. In contrast to the corresponding PM, the SD significantly enhanced the dissolution rate of IBU. As shown in the dissolution profiles, in the first 5 min, 60.0% and 83.7% of the IBU in the Kollidon CL and Kollidon CL-SF SDs was dissolved; these values are 15.9 times and 22.3 times those of pure IBU and the corresponding PM, respectively. Moreover, the selection of the carrier during SD preparation was also critical. In this study, Kollidon CL-SF produced a more remarkable improvement in the dissolution rate of IBU than IBU-CL; thus, the cumulative percentages of IBU in the IBU-CL-SF and IBU-CL SDs reached 94% and 80%, respectively, within 15 min. Considering all of the above results, the IBU-CL-SF SD was the optimal choice for improving the solubility and in vitro dissolution of IBU. 

Release kinetic modeling was conducted by using DDSolver software [21], including zero-order, first-order, Weibull, Makoid-Banakar, Peppas-Sahlin, and Korsmeyer-Peppas [22,23,24], as is shown in Table 2. The release profile of IBU and IBU-CL-SF SD were fit into a first-order kinetic model, whose equations were $F=57.736\times \left(1-e^{-0.009t}\right),r^{2}=0.9915\;\mathrm{and}\;F=90.550\times \left(1-e^{-0.862}t\right),r^{2}=0.9980,$ respectively. Among the following equations, the Makoid-Banakar equation was the best one for IBU-CL SD ($F=39.238\times t^{0.251}\times e^{-0.004t},\;r^{2}=0.9906$), IBU-CL PM ($F=8.811\times t^{0.455}\times e^{-0.004t},\;r^{2}=0.9969)$ and IBU-CL-SF ($F=19.688\times t^{0.244}\times e^{-0.002t},\;r^{2}=0.9844)$.

### 3.4. In Vivo Pharmacokinetic Evaluation

**Figure 6:**
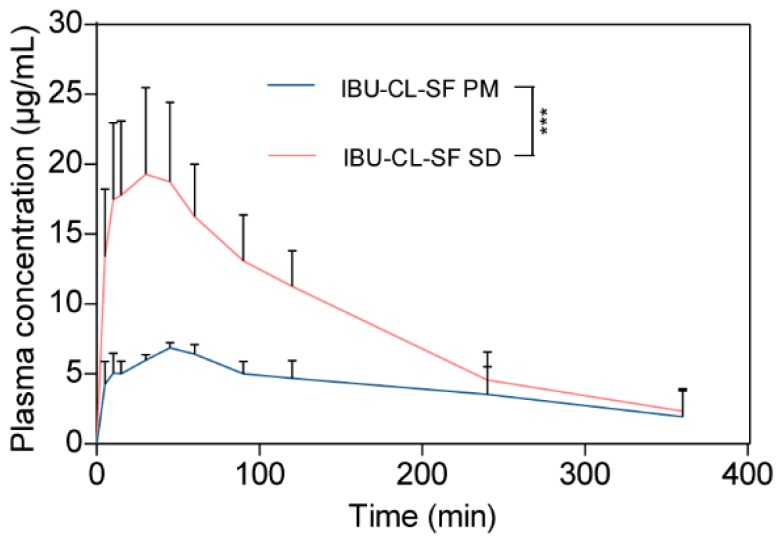
The profiles of mean plasma concentration-time. (Mean ± SD, *n* = 5, biological replicates). Statistical significance is represented by *** *p* < 0.001.

The profiles of the mean plasma concentration versus time for the IBU-CL-SF PM and IBU-CL-SF SD after administration are shown in Figure 6, while Table 3 shows the pharmacokinetic parameters of the IBU-CL-SF PM and SD systems.

After oral administration, IBU was quickly detected in plasma, and the maximum concentrations C_max_ of IBU-CL-SF PM and IBU-CL-SF SD were rapidly achieved, with values of 6.98 ± 0.18 within 48 min and 20.6 ± 5.4 within 33 min, respectively. Compared with those in the IBU-CL-SF PM, the C_max_ value of IBU was 2.95 times higher, and T_max_ decreased by 15 min in the IBU-CL-SF SD. These phenomena may result from the enhanced solubility and improved dissolution rate of IBU in the IBU-CL-SF SD during in vitro release.

All these results demonstrated that the oral bioavailability of IBU was significantly enhanced in rats owing to the increased dissolution amount and rate of IBU in the IBU-CL-SF solid dispersion prepared by the SCF method.

### 3.5. Drug–Polymer Interactions in Molecular Docking Simulation

**Figure 7:**
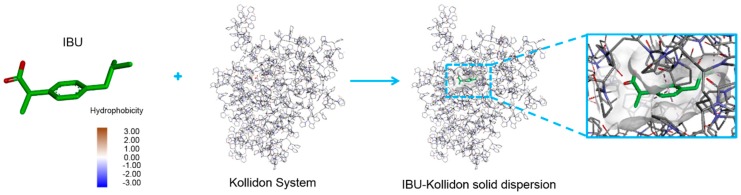
Docking conformation of ibuprofen complexed with polymers. Ibuprofen is shown in the 3D structure representation (left). Three close-up views of integral and local conformations relevant with the polymer, named Kollidon System and IBU-Kollidon solid dispersion, are displayed with the molecule and polymer shown as stick.

Kollidon affects the dispersibility of the drug and the particle size. Small particles can ensure uniform distribution of the drug and easily form a water-soluble complex, accelerating drug dissolution and improving bioavailability. The sizes of Kollidon CL and Kollidon CL-SF are 110 to 130 μm and 10 to 30 μm, respectively. As shown in the literature [25], binding of the drug to Kollidon was simulated by 1-methyl-2-pyrrolidone (Etp), which is similar in structure to the Kollidon unit. Here, as shown in Figure 7, the interactions of IBU with Kollidon systems were represented at the molecular level with both the polymer and molecule in three dimensions. Generally, the polymer effectively undergoes a hydrophobic interaction with the drug; from a thermodynamic point of view, a negative free energy (△G < 0) suggests a relatively stable system, whereas a positive △G indicates that an unstable system was formed when the molecule complexed with the polymer. The binding energy (△G) obtained in the molecular docking simulation, which was −5.71 kcal/mol, was used to evaluate the interactions between IBU and the polymers.

## 4. Discussion

**Scheme 2:**
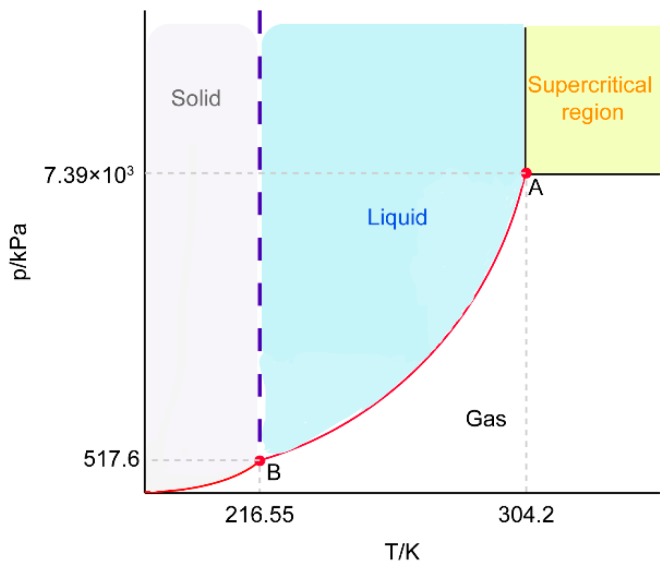
Phase diagram of CO_2_. A: Critical point; B: Three-phase point.

SCF technology, an experimental friendly process, minimized the particle size distribution, damage by shear forces, etc., in comparison with conventional approaches. It is categorized by a rapid expansion of supercritical solutions, supercritical antisolvents, gaseous antisolvents, particle formation from gas-saturation, and so on [26]. Among the processes above, SCF technology using scCO_2_ was greatly advantageous, since scCO_2_ is easier to remove from the system and the limited amount remaining would not be dangerous to patients. As shown in Scheme 2, point A is the critical point of CO_2_ (304.2 K, 7.39 × 10^3^ kPa), and when the system is above the critical pressure and temperature, the interface between the gas phase and the liquid phase disappears; this is called the supercritical state. IBU is a crystalline drug. The pressure of supercritical fluid has a great influence on the solubility of IBU. The density of CO_2_ increases as the pressure increases, and the ability to dissolve substances is proportional to its density. Therefore, the solubility of IBU was improved significantly. The XRD results suggested that the crystal peak of the drug-loaded-carrier disappeared, or the large amount of the carrier masked the peak of the crystalline drug. Therefore, we further adopted DSC characterization and molecular simulation to prove that the drug can be spontaneously loaded in the carrier to form a stable system (△G < 0). IBU, indeed, converted to amorphous, which is beneficial to the dissolution of the drug [27,28,29]. In the supercritical CO_2_, hydrogen bond dimers formed between the molecules of IBU. The high fluid density caused the dimerization balance between the monomers to shift, which weakened the bonding and increased solubility. In this process, IBU-CL-SF SDs were prepared at the same supercritical state (40 °C) but with different reaction times, pressure, and drug-to-carrier ratio. The results indicate that the cumulative percentage of the drug released with the extension of the three conditions. Prolonging the reaction time was preferable to obtain ideal solid dispersions, since the drug and carriers were more thoroughly dissolved and dispersed in scCO_2_. The cumulative percentage of drug release in IBU-CL-SF SDs increased with the rise of preparing pressure. This could be attributed to the fact that IBU in scCO_2_ possessed higher solubility under higher preparing pressure, resulting in the more thorough interaction between drug and carriers to obtain desirable solid dispersions. Considering the comprehensive factors, optimized conditions were determined. In addition, IBU has a relatively low melting point and boiling point. This makes IBU easy to sublimate and is also the reason for its high solubility. Solubility of poor water-soluble drugs is significant for enhancing oral bioavailability. Therefore, the preparation of ibuprofen solid dispersion by a supercritical fluid static reaction method is a very feasible method. We believe that Kollidon system SDs prepared by SCF technology will potentially become a carrier with good biocompatibility and high drug delivery efficiency.

## 5. Conclusions

IBU SDs were formulated with Kollidon CL and Kollidon CL-SF carriers in SCF media. The obtained results suggest that IBU-Kollidon CL-SF solid dispersion prepared by SCF technology markedly improves in in vitro and in vivo performance. Moreover, a molecular docking simulation showed that intermolecular interaction occurred and also suggested a relatively stable system. Using SCF technology combined with a Kollidon system to prepare solid dispersions is a promising approach to improving the absorption and oral bioavailability of water-insoluble drugs. We hope our study may open a new window for designing different formulations to improve bioavailability in the future.

## Figures and Tables

**Table 1:** Cumulative percentage of drug release from carriers at different conditions (reaction pressure, reaction time, drug-to-carrier ratio).

Group	Pressure (MPa)	Reaction Time (h)	Drug-to-Carrier Ratio	Cumulative Percentage of Drug Release (%)
1	20	6	1:5	73.21
2	20	12	1:5	80.44
3	20	18	1:5	87.90
4	15	18	1:5	80.58
5	25	18	1:5	91.05
6	20	18	1:1	77.45
7	20	18	1:3	91.45

**Table 2:** Release rate constants and r^2^ coefficients obtained from drug release profile based on kinetic equations.

Equations	IBU-CL SD	IBU-CL-SF SD	IBU	IBU-CL PM	IBU-CL-SF PM
Zero-order F = C + kt	F = 51.441 + 0.376t r^2^ = 0.2997	F = 71.947 + 0.266t r^2^ = 0.0875	F = 2.131 + 0.332t r^2^ = 0.9769	F = 16.276 + 0.369t r^2^ = 0.7279	F = 25.417 + 0.288t r^2^ = 0.5396
First-order F = a × (1 − e^−kt^)	F = 78.064 × (1 − e^−0.366t^) r^2^ = 0.9474	F = 90.550 × (1 − e^−0.862t^) r^2^ = 0.9980	F = 57.736 × (1−e^−0.009t^) r^2^ = 0.9915	F = 45.904 × (1−e^−0.076t^) r^2^ = 0.9328	F = 44.493 × (1 − e^−0.247t^) r^2^ = 0.9328
Weibull F = 100×{1 − e^[−(t^β) / α]^}	F = 100 × {1 − e^[− (t^0.294) / 1.873]^} r^2^ = 0.9630	F = 100 × {1 − e^[− (t^0.278) / 0.934]^} r^2^ = 0.9742	F= 100 × {1 − e^[− (t^0.869) / 128.345]^} r^2^ = 0.9912	F = 100 × {1 − e^[− (t^0.410) / 9.318]^} r^2^ = 0.9858	F = 100 × {1 − e^[ −(t^0.248) / 4.333]^} r^2^ = 0.9794
Makoid-Banakar F = k × t^n^ × e^−bt^	F = 39.238 × t^0.251^ × e^−0.004t^ r^2^ = 0.9906	F = 66.349 × t^0.135^ × e^−0.003t^ r^2^ = 0.9870	F = 0.637 × t^0.936^ × e^−0.003t^ r^2^ = 0.9910	F = 8.811 × t^0.455^ × e^−0.004t^ r^2^ = 0.9969	F = 19.688 × t^0.244^ × e^−0.002t^ r^2^ = 0.9844
Peppas-Sahlin F = k_1_ × t^0.5^ + k_2_t	F = 26.104 × t^0.5^ − 1.821t r^2^ = 0.8292	F = 34.669 × t^0.5^ − 2.603t r^2^ = 0.3378	F = 1.237 × t^0.5^ + 0.223t r^2^ = 0.9875	F = 8.968 × t^0.5^ − 0.404t r^2^ = 0.9967	F =12.862 × t^0.5^ − 0.794t r^2^ = 0.8805
Korsmeyer-Peppas F = k × t^n^	F = 45.960 × t^0.140^ r^2^ = 0.9294	F = 72.461 × t^0.064^ r^2^ = 0.9460	F = 1.020 × t^0.766^ r^2^ = 0.9895	F = 11.220 × t^0.329^ r^2^ = 0.9771	F = 21.366 × t^0.192^ r^2^ = 0.9762

**Table 3:** Pharmacokinetic parameters of IBU-CL-SF PM and SD systems.

Parameters	IBU-CL-SF PM	IBU-CL-SF SD
C_max_ (mg/L)	6.98 ± 0.18	20.6 ± 5.4
T_max_ (min)	48 ± 6.7	33 ± 12.55
AUC_(0–t)_ (mg/L × min)	1476.4 ± 411.7	3203.7 ± 450.9
MRT_(0–t)_ (min)	140.5 ± 30.8	115.7 ± 23.8
